# Stakeholder Perspectives to Support Graphical User Interface Design for Children with Autism Spectrum Disorder: A Qualitative Study

**DOI:** 10.3390/ijerph18094631

**Published:** 2021-04-27

**Authors:** Betania Groba, Laura Nieto-Riveiro, Nereida Canosa, Patricia Concheiro-Moscoso, María del Carmen Miranda-Duro, Javier Pereira

**Affiliations:** CITIC, Research Group TALIONIS, Faculty of Health Sciences, Universidade da Coruña, 15071 A Coruña, Spain; b.groba@udc.es (B.G.); nereida.canosa@udc.es (N.C.); patricia.concheiro@udc.es (P.C.-M.); carmen.miranda@udc.es (M.d.C.M.-D.); javier.pereira@udc.es (J.P.)

**Keywords:** autism spectrum disorder, children, graphical user interface, qualitative approach, digital support, assistive technology, activities of daily living

## Abstract

The development of digital supports for people with autism has increased considerably in recent years. Technology designers and developers have interpreted the needs and learning styles of people with autism in different ways. As a result, there are generic, non-specific or heterogeneous guidelines for the design and development of technology for people with autism. This study aims to identify and describe the recommended elements to support graphical user interface design for children with Autism Spectrum Disorder (ASD), considering the stakeholders’ perspective, engaged in a computer application development. A qualitative, longitudinal, multicentre study was carried out. A sample of 39 participants belonging to four groups of stakeholders participated: children with autism, family members, professionals with experience in the intervention with children with autism, and professionals with expertise in the design and development of assistive technology. The techniques used to formalise the collection of information from participants were semi-structured interviews and observation. MAXQDA 2020 software (Verbi Software, Berlin, Germany) was used to analyse the data. The result is a guide with suggestions to support an interface design that emerges from the stakeholder perspectives. This study provides useful information to offer alternatives for children with ASD and facilitate the understanding of daily life.

## 1. Introduction

Autism Spectrum Disorder (ASD) is a “heterogeneous, neurodevelopmental condition that persists throughout life” [1] (p. 1). Autism originates in childhood and is characterised by the presence of persistent difficulties in communication and social interaction and repetitive and restrictive patterns of behaviour, activities, and interests [2]. People with autism present with symptoms that have a significant impact on daily functioning, which is recognised as a diagnostic criterion [2].

The prevalence of ASD is not clear and different studies conclude different results. It is now generally accepted that approximately 1% of the population has ASD, both children and adult [2] (p. 55). The latest data from the U.S. Department of Health and Human Services Centres for Disease Control and Prevention (CDC) point to an increasing trend in prevalence and establish that 1 in 54 children has autism [3].

Information and communication technologies (ICT) are emerging as tools with great potential to facilitate the daily life of children with autism. People with autism report high levels of use of technology in daily life [4,5,6,7]. The predilection, interest, and motivation of most children with autism for technology have been widely reported in research since the 1970s [8,9]. Underlying reasons for this predilection may include the characteristics of technology as a controlled, structured, and predictable environment, and human-technology interaction without demands for social interaction. In a recent survey conducted in the United Kingdom, Spain, and Belgium, it has been reported that children with autism have access to and use different technologies for their everyday leisure [4]. Likewise, children and adolescents with autism present high competence for the handling and use of technology [4].

Previous study results support the effectiveness of using technology in children with ASD for learning new skills [10]. Several systematic reviews recognised the effectiveness of technology for the acquisition of academic skills [11,12,13], communication [14,15], social interaction [16], emotional learning [17] and independence in performing activities of daily living (ADLs) [18].

In the good practice guidelines for autism intervention, technology aided instruction intervention (TAII) is recognised as an evidence-based practice recommended for intervention in children, youth and young adults with autism [19]. Although technology is considered an emerging and promising tool, it is not yet consolidated, due to the limitations of studies, wide variability of participants with ASD, and difficulties in generalising learning to the daily life of this population [1,10]. Recommended as good practice is that any intervention for people with autism should have an approach focused on each person’s strengths [1]. Thus, evidence-based practice, and motivation towards technology in children with autism may be promising aspects for intervention, with these supports.

Assistive technology includes hardware devices and applications or software. Digital supports for people with autism are defined as “any electronic item/equipment, application, or virtual network that is used to intentionally increase, maintain, and/or improve daily living, work/productivity, and recreation/leisure capabilities” of people with autism [20] (p. 2). Digital supports are devices, applications or products that have been specially designed and developed to promote participation in daily life [21,22].

Tablets and personal computers are the most widely accessed and used hardware by children with autism [4]. In addition, there are experiences based on the use of other mobile devices, video modelling, robots, sensors, virtual and augmented reality [18,23].

Initially, software resources were limited, requiring use of generic or commercial software that normally presented considerable additional economic cost. In the last two decades, software developed in this area has increased in such a way that it has been necessary to develop initiatives to try to filter and select the most appropriate applications for each person with ASD [24,25]. In Spain, the APPYautism application provides contrasted information about approximately 410 apps for people with ASD [26].

The development of digital supports for people with autism has increased considerably in recent years [22]. Software developed to facilitate daily life and for learning specific skills through serious games is highlighted [27,28].

This increase in technological development and offer have not been accompanied to the same extent by research studies on the effectiveness of these supports, nor by exhaustive studies on their design and development process [22]. Although technology developers expressed a clear interest to design and develop programs that met the needs of this population, there is a high variability of design approaches. Technology designers and developers have interpreted the needs and learning styles of people with autism in different ways. The rationale for designing technological solutions in one way or another has not often been made explicit. As a result, there are generic, non-specific or heterogeneous guidelines for the design and development of technology for people with autism.

For years, it has been suggested that people with autism have specific strengths and needs that should be considered in the design and development of technology: special interests, visual processing, differences in sensory perception, attention to detail, routine affinity, and a predilection for sounds, images and characters [29,30,31,32].

Furthermore, different authors concur that there are several special requirements software designed for people with ASD must meet: high level of customisation of the technology, use of concrete visual information, consistent structure for predictability, adequate feedback and reward, dynamic stimuli, clear audio and engagement of end-users and stakeholders in the process [22,31,32,33,34,35,36,37,38,39,40,41,42,43].

In previous years, some qualitative or mixed-method studies have focused on understanding the perspective of the different agents involved in the use of technology in the field of autism; specifically people with ASD, family members and ASD-relevant professionals [41,42,44,45,46,47,48,49]. The focus has been on exploring the meaning given by end-users and stakeholders to promote inclusive, person-centred and evidence-based technology design and development practices. This new approach addresses the integrating issues that had been ignored, such as the perspectives of people with autism themselves, their families and professionals, and the pedagogical, motivational and therapeutic theories, so that people with autism can get engaged, learn and participate to a greater extent with the use of digital supports.

Despite these efforts, the results are generic and clear examples of how to apply these principles in technology design and exemplifies that development still needs to be further studied.

This study aimed to capture the perspective of stakeholders engaged in the development of a computer application for children with ASD in order to identify and describe the recommended elements to support the graphical user interface design.

## 2. Materials and Methods

### 2.1. Design

A qualitative, longitudinal, multicentre study was carried out, based on a phenome logical approach.

The knowledge extracted from a qualitative approach enables to deepen the analysis of occupation and daily life from the experiences and perceptions of the people who participate and get involved in them [50,51]. This methodology was chosen to collect the perceptions, experiences and decision-making of the people engaged in the design and development of a computer application for children with autism.

### 2.2. Participants and Settings

A sample of 39 participants belonging to four groups of respondents participated (see Table 1), meeting the following criteria:

- 1st Group: professionals with experience in the intervention with people with ASD.

The study involved 20 professionals recruited from centres of direct care for people with ASD, such as two schools of special education for children with ASD diagnosis, an ASD-specialised clinic for psychological intervention, and an association that provides support for adults with autism and their families. The selection criteria for these professionals were: (a) a degree in education sciences or health sciences or representing the interests of organisations that serve people with ASD; and (b) ability to prove work experience of at least one year in the intervention with people with ASD. Finally, a heterogeneous group was obtained, made up of three representatives of organizations, three psychologists, 11 schoolteachers (seven teachers specialized in therapeutic pedagogy), one social worker and two speech therapists.

- 2nd Group: professionals with experience in the development and design of technology for people with disability.

The project involved 13 professionals recruited from two centres that made use of the technology applied for social and/or health context. Participants also met the following criteria: (a) a degree in the field of technology or health sciences or promoting social projects; and (b) ability to prove work experience of at least one year in the design, development and/or testing of digital technology related to the health and quality of life for people with disability. The group involved the collaboration of four experts in the implementation of social projects, one doctor, five engineers in computer science and three occupational therapists.

- 3rd Group: family members of people with ASD.

Participants were three direct relatives of people with ASD and, in turn, they were part of the organizations for people with this diagnosis.

- 4th Group: children with ASD.

In the final phase, three children (two boys and one girl) participated, aged between 10–13 years. All participants were selected from a special education centre and met the following criteria: (a) a diagnosis of ASD; (b) persistent difficulties in daily functioning; and (c) not having used the specific software prior to testing. Marina, Tadeo and Bruno (pseudonyms) did not have functional verbal language. They used visual supports and signs for communication.

### 2.3. Procedure

**Figure 1:**
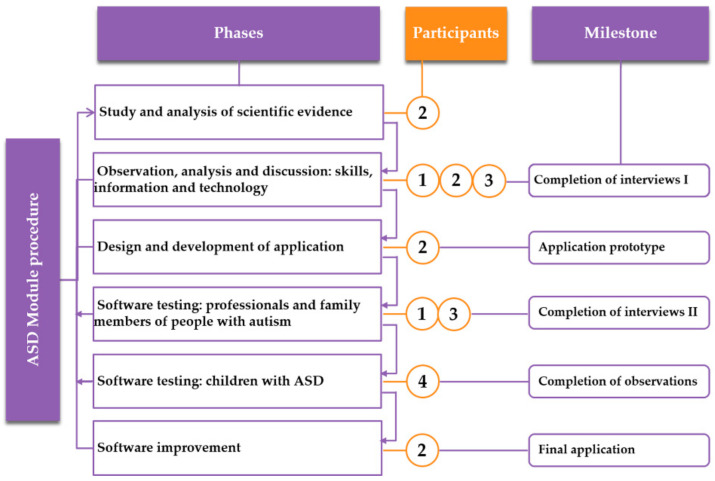
Study procedure (phases, participants, and milestones).

This research was based on a user-centred design and followed an iterative procedure (details of the procedure can be found in Figure 1).

### 2.4. ASD Module: Software Tool

The ASD Module (Fundación Orange, and IMEDIR, A Coruña, Spain) was the tool used during the study and is a free technological application that is made up of a set of virtual keyboards (or adapted interfaces), digital schedules and activities, especially designed and tested by and for this study group and included in the In-TIC PC software (Fundación Orange, and IMEDIR, A Coruña, Spain) [52]. The software is available for the Windows operating system and was implemented with the Visual Studio development tool, .NET environment, C# programming language and Windows Form technology. Other materials used for content development were the Interactive Books Multimedia (LIM, according to the Spanish acronym) software (Macías, F., Lugo, Spain) [53] and Aragonese Portal of Augmentative and Alternative Communication (ARASAAC, according to the Spanish acronym) pictograms [54].

**Figure 2:**
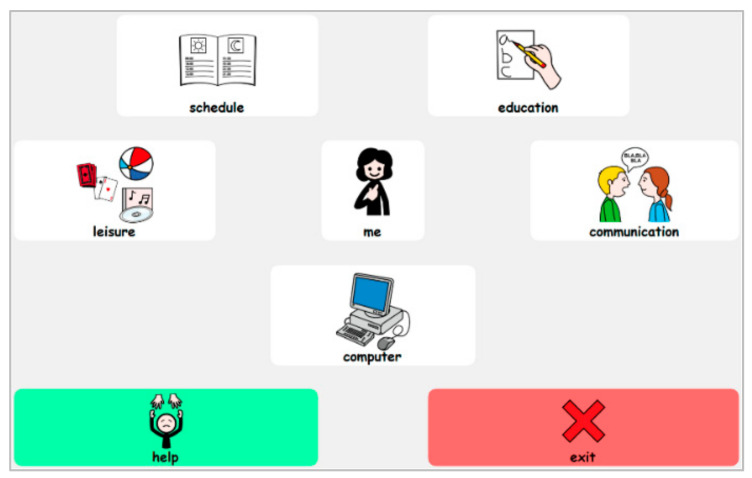
The main screen of the ASD Module.

The ASD Module is based on the perspective of considering the person with autism as the central axis, and the activities of their life interests surrounding this perspective. To provide an example that can be adapted and customised to other people, consider the case of Arancha, a girl with ASD. When analysing the main screen (Figure 2), Arancha is observed in the central part and, around her, the activities in which ICT can support her in daily life: schedule, education, leisure, communication and computer access [55].

Schedule. This section refers to the user’s basic daily activities. It was designed to access timetables, schedule and activity sequences easier in the goal of organising a person’s day and activities.

**Figure 3:**
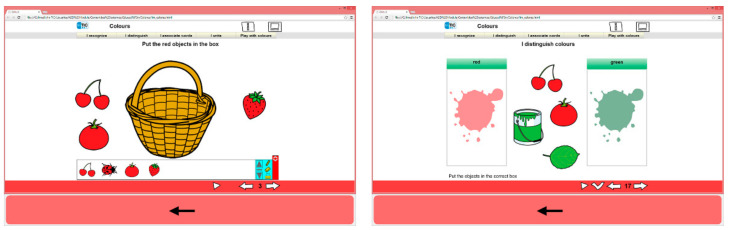
Examples of LIM activities using colours for the Education section.

Education. This section includes activities necessary for learning and participation in the school environment. Its contents refer to five basic categories: colours, numbers, letters, parts of the body and feelings. Moreover, dynamic activities have been designed on the same line with the LIM system (see Figure 3).

Leisure. This section refers to leisure activities and includes resources classified as games, stories and documents regarding people with ASD.

Communication. This section contains basic communication keyboards. Pictograms and/or pictogram writing are used, and when combined with speech synthesis resources, they stimulate communication of basic requests and needs. In addition, other keyboards favour the narrative discourse and development of questions encouraging a person’s social participation.

Computer access. This section refers to access to several conventional programs of Windows operating system, in which both access and use are simplified. The programs used are: Wordpad (text editor), Microsoft Paint (drawing program), Windows calculator and Windows Media Player (Windows 7, Windows Vista and Windows 8 compatible versions, Microsoft Corporation, Washington, DC, USA).

### 2.5. Information Collection Techniques

The techniques used to formalise the collection of information from the different groups of participants were semi-structured interviews and observation.

#### 2.5.1. Semi-Structured Interviews

The interviews enabled us to explore the meaning of the use of technology in the daily lives of children with autism and the perceptions regarding the essential design aspects to be considered in the development of the interface, as well as the contents included in a technological solution for children with autism. The interview guide was the same for all participants (it can be found in Appendix A).

Individual face-to-face interviews were conducted with adult participants in two rounds. Interviews between 30–60 min in duration were conducted by the first author individually with each participant. In the second interview, a computer with the ASD Module prototype was used to allow participants to test the software. Semi-structured interviews were conducted in a room provided by the participants’ reference centres.

The interviews were audio-recorded, with the consent of the participants. A transcript of the interviews was then made and the identities of the participants coded. The information that might allow identification was anonymised. Subsequently, the audios were deleted.

#### 2.5.2. Observation and Usability Test

Marina, Tadeo and Bruno tested the software, once a prototype application with stable behaviour was obtained.

The following participants were present at these tests: the child’s reference teacher, a speech therapist, a teacher specialising in therapeutic pedagogy and the first author (an occupational therapist). Each child’s teacher was the only one who provided instruction and support. The duration of the sessions was approximately 20–30 min.

Each child was encouraged to sit in front of the laptop on which the stable prototype of the ASD Module was installed. In the first session, the objective was for each child to explore the software freely. In the second session, each child was asked to perform the following actions with the ASD Module: make a request (Communication); complete the sequence of activities for that day (Schedule); select the requested education activity (Education I); complete at least two virtual education activities (Education II); choose a video (Leisure) and write his/her name in the text editor (Computer Access). Also, the use of virtual keyboards in a functional way (Virtual Keyboards) was assessed.

During the second observation, the above items were scored with a single response with a single score resulting from the agreement among the four observers. Each item was scored on a scale of 0–3: 0 = child does not perform the task; 1 = child performs the task with support (mainly physical support); 2 = child performs the task with reinforcement (mainly verbal prompting), and 3 = child performs the task independently.

In addition, qualitative aspects were collected regarding the observation of each child’s interaction with the software interface.

### 2.6. Data Analysis

MAXQDA 2020 software [56] was used to analyse the qualitative data. An inductive thematic analysis was used, with the categories that explained the phenomenon emerged directly from the information in the interviews and observation [57]. The phases described by Braun & Clarke in 2006 were followed for the thematic analysis: familiarising with the data, generating initial codes, searching for themes, reviewing themes, defining and naming themes, and producing the report [58].

The coding process was carried out by four authors (B.G., L.N.-R., P.C.-M. and M.d.C.M.-D.) until it reached data saturation. First, the interviews were transcribed and anonymised by the first author. Second, the transcripts were carefully read by the four authors involved in the analysis process. Third, individual codes were searched for, labelling text units that were relevant to explain the phenomenon of study. In this phase, 107 codes were identified. After the search, definition and redefinition of the themes and the analysis among the four authors, the 107 codes were grouped into 47 sub-themes. Finally, 10 final themes were obtained, which were drafted and revised in the final report.

## 3. Results

### 3.1. Results from Interviews

The results arose from the inductive thematic analysis of the interviews with the participants. The description of each theme was accompanied by verbatim records from participants selected for their relevance to reflect aspects of each theme. The involvement of each category in the decision-making for the design and development of the ASD Module was also explained. A summary of the themes, sub-themes, and implications for software design can be found in Appendix B.

#### 3.1.1. The User Has the Possibility to Customise All Relevant Aspects

The participants indicated that it is important to have an example depicting the possibilities of the software. In general, they indicated that the ASD Module is an interesting example to facilitate professionals and families’ work, but that it should allow adaptation and personalisation for each child (see verbatim quotes for participants 17 and 18).

*“Considering that it is as a generic model, I would not add anything else. Only later, when you have the person you are going to work with, should you have to make several changes to focus on their preferences, their interests, and their person”*

(P17)

*“I think that will be determined by the person who uses it. When it is used with the person, it will be the person who will determine. Well, I have to strengthen this part, or I have to eliminate, expand [the software options]... Once it is used, I think it will be the person who will determine the needs, the interests”*

(P18)

In the speeches, constant difficulties were perceived in establishing statements that apply to all people. The word “depends” appeared emphasised numerous times: *“I am going to be telling you that all the time. It depends, it depends, it depends, it depends” (P6)*.

It was directly linked to the participants’ references to diversity and conviction that each child is unique, regardless of the diagnosis of autism: *“It is complicated to talk about something... for me, huh? It is complicated; what I was telling you before: it is so variable from one person to another, that I do not dare to do more... No, I could not tell you...” (P7)*.

The interventions aimed at people with ASD should be customised and individualised. The conclusion was that any technology should support customisation. Therefore, even within the specific software, adapting certain aspects to each child’s needs and potential is essential.

The ASD Module refers to customisation as a key point of the software design. Thus, all contents, images, letters, audio elements, and colours can be adjusted and customised, depending on the child’s preferences.

#### 3.1.2. The Software and Its Contents Are Based on a Person’s Abilities, Needs and Interests

All participants indicated that technology and technological solutions should respond to people’s everyday needs. In the context of children with autism, this aspect is considered essential. Digital supports for children with autism should be based on their learning styles, and from a user-centred perspective, focus on their abilities, needs, interests, and motivation.

In order to capture these aspects, end-users and stakeholders must be involved in the design and development process. Children with ASD, family members, and people within their immediate surroundings have been excluded from the technology design and development process. However, the participants described this process as an exciting and successful experience.

The participants in this study highlighted that the contents they considered most relevant to be developed in the project were related to the following areas: communication, leisure/play, time and activities management and education. These skills are reflected in the ASD module’s contents in the schedule, education, leisure, communication, and computer access sections.

The interviews described the importance of considering the motivation of children with autism to become engaged and participate in activities. For instance, participant 19 explains: *“We have many ways to reach them in a motivating way. People who have difficulties in terms of social understanding and understanding what is going on around them need a different form of motivation, and you can recreate a visual world, a whole world adapted to their way of thinking. Moreover, the truth is that all these programs are wonderful. (…). It is a new field that I think is going to help them a lot” (P19)*.

Detailed is that children with autism usually show interest and motivation for technology and, in many cases, they show high levels of competence in its use. Technology is perceived as a tool with a strong potential to facilitate this population’s lives: *“[The computer] is an extreme interest for him. Because here we have to talk about motivations, which is fundamental. It is an extreme interest” (P9);” And... well, also to say, not in all cases, but in the most, a computer is a tool that attracts their attention. So, it is beneficial for them” (P17)*.

Interestingly, the patterns of interest displayed by people with ASD have been described as atypical. However, the participants showed that people with ASD may benefit from integrating their interests into the intervention, rather than discarding them.

During the development of the software, some general topics were considered for the activities that should be customised to include each person’s interests.

#### 3.1.3. The Design of the Interface Is Simple, and the Information Displayed Is Simplified

Participants agreed that process information in children with ASD is different, and their attention and concentration difficulties may occur due to the predisposition to “a detail-focused” information processing style.

The interface should prevent children’s distractions and help them focus on the main elements of the screen, which is why it is essential to reduce visual complexity.

In the speeches, there were references to the interface, that it should contain little information and large size; present a grid layout or, at least, a consistent distribution among the different screens; and a left-right organisation (see quotes from participants 6, 9, 25 and 8).

*“We, let us see, we are very interested in the structure. (…). The structure, including that of the program, that the environments speak for themselves, that the children can manage by themselves...”*.

(P6)

*“Everything [should be] very clear and very intuitive at the moment, without much information; in the case of these children with more difficulties”*.

(P9)

*“Logically, it cannot be one thing too much, with much information, because they will get lost... The more information you give on the screen, they will get lost, it is obvious. (…). So, little and straightforward”*.

*“Concrete information and always in four or five buttons. It means that there should not be too much saturation, a saturation of images”*.

(P25)

*“This is a bit like writing and I always go from left to right”*.

(P8)

The ASD Module was organised using dynamic interfaces called *keyboards*. For the aspects mentioned above, the ASD Module keyboards were configured, always using the whole display area when possible, to reduce and simplify the information contained therein.

In addition, the keyboard organisation was developed in a grid pattern so elements could take up individual spaces at regular intervals. The position of the elements on the interface was relevant for the user, who should have been able to locate them easily. Thus, they were organised along the left-right axis. It was necessary to consider the work zone that end-users occupied and how they analysed the elements’ organisation, since there were implications of head and eye movements, attention and concentration skills, and laterality acquisition. Therefore, the buttons’ arrangement was adapted to the person by placing the buttons or pictograms along a vertical sequence, and once people increased their skills, a mixed (left-right) or horizontal distribution was used.

#### 3.1.4. Use of Images to Display Information

People with ASD have several abilities in common. Participants concurred that one of the abilities that many children with ASD may share is the ability to process visual information more effectively than verbal information (as participants 34, 35 and 23 explain in their interviews). The results of different information sources suggest that people with ASD have a greater predisposition than the rest of the population to visual, rather than language, processing in order to understand the meaning of sentences.

*“Because my son can take the belt out of the car and say to me: ‘Look, stop, stop... Where are we going?’. However, if he in the morning stands up, and takes a picture of his grandmother’s house, of a spa, of a walk along the beach, of a shopping basket, of a walk, of a coke…; any minimum indication (…); he already knows where he is going; that day, he already has it organized”*.

(P34)

*“Let us see; life is a continuous change for someone who knows how to decode information. However, they (children with autism) do not have codes. They do not have the same code as us. In other words, if we do not move in the same code, I have to show them their code, what they know how to read and what they know how to internalize. What is it good for? They do not understand. What is it good for? Supports have to be very logical: visual keys in their daily life”*.

(P35)

*“I think so because it is what I was telling you before, it is like very... visual. Everything that computers are, and all this. Moreover, they learn much better through the visual channel than through words, for example. You can tell them a huge story and they might understand it much better if you accompany it with pictures or just with images. So, computers give us this possibility”*.

(P23)

Visual support is commonly used to promote the development of different skills in children with autism and provide supplementary information with greater significance. Due to these reasons, all of the material of the ASD Module was based on the use of images.

#### 3.1.5. The Images Convey the Meaning of the Real Element

Professionals and family members indicated that they use ARASAAC pictograms because of their iconicity with the real element they represent: *“[The ARASAAC pictograms] are very iconographic drawings” (P9).* In Spain, ARASAAC pictograms are usually used in interventions with people with ASD, especially in recent years.

Those professionals with more years of experience indicated that centres started using Picture Communication Symbols (PCS), but were later replaced by ARASAAC pictograms. This change was mainly due to the transparency of the images, their adaptation to the Spanish context, and the fact that they were free of charge: *“No, before there were the Boardmaker ones. What happens is that the Boardmaker pictograms, some of them, were a little bit too short, and ARASAAC has, they are more updated... They are more explicit, more fixed... I do not know, more concrete” (P5); “Others, the others [PCS symbols] are sometimes so schematic that it could be that as it could be any other... It does not speak in itself. The drawing is not speaking. Nevertheless, they have associated that this strange form means that” (P16)*.

On the other hand, interviewees described that children with ASD use, remember, and mostly associate sets of more realistic and representative symbols than those that are abstract. They may prefer realistic pictures, which are familiar with their daily life.

For these reasons, the In-TIC software included 9500 pictograms and 950 pictures from ARASAAC [54], which were transferred for distribution. Both sets are available in colour and are customisable in each of its aspects.

#### 3.1.6. The Use of Images Allows Users to Adapt According to their Level of Visual Cognition

The participants acknowledged in their speeches that children with autism learn better through visual information. However, the generalised statement that all children with autism learn better using pictograms was an aspect of concern for study participants.

One point identified in children with ASD regarding understanding instructions is based on the assessment of the level of visual cognition of the person who will be using the program. This refers to the set of symbols (e.g., object, picture, pictogram) that the user able to recognise and associate with the real item it represents.

Some participants reported a possible progression of symbol acquisition, increasing the level of difficulty for their understanding: (a) objects with a meaning (e.g., a cup may mean breakfast or may involve miniature objects or parts of objects); (b) pictures (provided that the irrelevant information is removed therein); (c) pictograms (a higher level of abstraction is needed for understanding); and (d) written words. However, other participants stated that this possible hierarchy did not necessarily persist in all situations.

These issues had implications for the software design because, in the case of the ASD Module, even if some specific pictograms were used, it allowed users to customise the program using other symbol sets, their own pictures, or written words to suit their level of development: *“What about those who have to use a photo? They don’t understand with pictograms... (...). Because if I tell them: ‘Let’s go to grandma’s house’; I can’t use that grandma [pictogram]. I don’t need that grandmother; I need HIS grandmother... Can I put it in [the software]?” (P6); “They may need a real picture or pictogram... They may need a real picture, a pictogram, another pictogram, a drawing... They may need it...” (P6)*.

#### 3.1.7. The Image Is Accompanied by the Written Word

From the participants’ perspective, using a combination of visual and written information may be one of the most effective ways to teach this population. The information display could be performed according to each person’s level, however, regardless of the set of symbols used, it may be important to combine it with the written word.

The professionals working within the immediate surroundings of the children who participated in the project indicated that this aspect might be beneficial, even though the person with ASD had not acquired the literacy aptitude needed, due to the possible unconscious connection between the image and word: *“I prefer to always use it for, well, if they can acquire literacy later, then the word is already there” (P9)*.

In the interviews, participants stated that the word should be located at the bottom of the image. Based on their experience, this location allows children to focus on the image and have additional written information in a complementary way: *“We, we put the text below. (…). First, you, see the drawing, and [the written word] is like the caption, isn’t it, of a...? Sure, in any text that there is a picture, the text is underneath” (P12); “[You focus] first on the image and then on the word. Underneath, underneath. Although there, I think it also depends on the child you have or the child you work with” (P13)*.

The formatting aspects of the word were the subjects of differences among the participants. One group of participants suggested the use of capital letters and a print or serif type style: *“When it is capitalised, the simplest, the straightest; for example, I would put. The one we could see the clearest” (P20)*. However, another group of participants suggested the use of lowercase and a calligraphic-linked typeface: *“But, however, it was always said that in lowercase because the lowercase offers a form in the word that is more recognisable” (P7)*.

The reasons for choosing one format or the other depended on the literacy teaching methodology. In the final round, it was agreed that the format for the ASD Module would be lowercase and calligraphic-linked style (the suggested fonts were Comic Sans or Edelfontmed); however, customisation would be allowed.

The pictograms and word writing (at the bottom) were the primary sources of information in the software.

#### 3.1.8. Speech Synthesis Is Used to Facilitate Communication or as Reinforcement to the Command

Many people are sometimes hesitant regarding whether a technological device with speech synthesis should be used. They fear that its use may impair the further development of oral language or it may influence the user’s phonology of the existing language.

In the design and development process of the ASD Module, the participants generally recommended the use of synthesised voice for children with autism: *“To me, it [the synthesised voice] seems very natural. I think it’s fine” (P36)*.

The main benefits of using synthesised speech identified in this study are augmentative or alternative communication systems and auditory reinforcement of information. In both cases, it is reported that children tended to prefer software characterised by high levels of interaction, such as animation, sounds, and voice features. Despite the general recommendation of the use of synthesised voice for these purposes, consideration of the aspects related to the auditory hyper-reactivity of some people on the spectrum may be indicated, and this aspect can be customised, as reported by participants 13, 33 and 8.

*“There are hypersensitivities. There are some who, perhaps, it may seem to them a very dry tone, very, very... metallic; perhaps. Yes... Very imperative, you know? Very ‘agenda’ (she imitates synthesised voice). Maybe if you modulate [the synthesised voice] a little more, no?”*

(P13)

*“He [her son with autism] has oral language. But he can’t stand this kind of voice... He doesn’t like it. That brass tone, he can’t stand it. Because we had toys with that kind of voice and a kind of tablet but it repeated also in that singsong, and... he covered himself [pointing to the ears]”*.

(P35)

The interactive elements of the ASD module, called buttons, can perform several actions according to the selected activity. All elements included a voice command using speech synthesis. The speech synthesis messages were simple, specific, and short to avoid empty or irrelevant language: *“As long as they are short messages, which was what I saw [in the software], and simple and without too much... I imagine the tone of voice, no… If it’s pleasant it shouldn’t be a problem” (P32)*. Likewise, the messages made an impression of natural social interaction.

As described, speech synthesis was a useful resource to provide commands or improve communication for people with ASD. This resource can be customised if it is noticed that the end-user responds inappropriately. Also, it could be replaced by the digitised voice of someone familiar to the child: *“Sure, I would be a little bit in favour of putting the most common voice of the environment where they move... Is it worth it, for example, to go through the whole process of, that, recording with natural voice? You or me...? Recording ‘agenda’, ‘education’, the different words…, so that it sounds natural rather than synthesised? It will depend on the child” (P8)*.

#### 3.1.9. The Information Is Displayed in a Multimodal Way (Visual and Auditory) and Is Adapted According to the Sensory Style Preferred by Each Child

The participants’ perceptions of the single or mixed modality of information presentation have been different. On the one hand, the participants’ consensuses after interview analysis were that children with autism learn more easily if a combination of different stimuli is used (e.g., simultaneous presentation of visual and auditory information). These participants described numerous experiences based on the use of stimuli presented through different sensory channels to facilitate learning for children with ASD: *“Yes, yes. I, for example, when they look for a pictogram, we always reinforce it. Even with many of them, we use the sign, the pictogram, and the word, all at the same time. When I make the agenda with him, who is one of my students, when I tell him: ‘look for a school’, I make the sign, I support him visually with the sign. In other words, it is always good. And, in 90% of the cases, people probably use it both with the word and the pictogram. But there’s always that 10% of people...” (P11)*.

They also considered that technology has important potential to enable the display of both visual and auditory information.

On the other hand, some participants reported that the presentation of information perceived through different sensory channels could lead to distractions, comprehension difficulties, partial processing of information, or even hyper-reactive to stimuli. *“They have a sensory coding problem. (Imitated voice) ‘Please, if I’m seeing you, I can’t hear you.’ There are kids who can’t hear when they’re seeing. It’s mind-boggling; they can’t” (P6); “It’s really that two visual channels coming in, kills them. But since in life itself they have both, sighted and hearing, there might be a reinforcement there. Can we have one removed?” (P19)*.

This group of participants indicated that it was necessary to allow customisation of this aspect, depending on each child’s sensory preferences (see verbatims quotations from participants 6, 19 and 2).

*“I imagine it could be a further reinforcement. In addition to the visual, there is also the auditory. Why? It wouldn’t have to be incompatible. In fact, it’s the most natural thing, isn’t it? (…). It could be a customisable option. Take it away, when he already knew it”*.

(P2)

In the ASD module, the multimodal presentation for buttons was used to provide the information through the image, written word, and speech synthesis. Moreover, it offered the possibility to delete or add information to be processed through one or several channels according to the preferred sensory style of the person with ASD who uses it.

#### 3.1.10. The Background Colour Is Used to Facilitate Information Processing

The consensus of the study was that background colour is a key enabler for children with autism: *“I noticed that there were preferences and what I did notice is that colours help them to decipher. The basic colour” (P22)*. In practice, the role of colour as a facilitator of learning was considered, especially for gathering information by categories of meaning.

The participants recognised in their speeches that the emergence of the classification established according to the background colours of PCS^®^ Mayer Johnson commercial symbols (1990) created an opportunity to systematise teaching procedures for children with disabilities. This classification has been widely applied in practice, and it groups the differently coloured PCS pictograms according to the grammatical functions of the words represented.

Despite this, the use of classification was adapted to the needs of the centres. At times, the classification was partially implemented (see the explanation of P7, and P13). In some instances, it was replaced by the use of background colours, depending on the association of the words to specific conceptual categories (e.g., outings, ADLs, education).

*“Because as some of us use the Boardmaker, others, another; others, another; others, another... At one time, when we started, we thought we had to try in the centres of Galicia [for children with autism] to homogenise the use of a system because as the children went to camps or camps with one association or another, they could suddenly find themselves without language, right?”*

(P7)

*“Let’s see, we would have to do it. I categorise. I’m talking about my classroom. Time and people’s names; that is, I have yellow backgrounds in everything that refers to people, and everything that refers to time is in blue. And then I work with white”*.

(P13)

However, it was clear to the participants that, regarding the interfaces of programs for children with ASD, colour is a source of information that may turn into a learning facilitator or barrier. That is why background colours should always be used for a specific purpose, that is, to facilitate the organisation of information: *“I imagine that what may be important is that the option they are going to choose stands out, that it is really more striking than the others” (P20); “Maybe what’s more important, is that the colours are used as a clue and in a consistent way” (P15)*.

Also, they recommended a contrast between the image and background, but that the colour tones should be soft (avoiding bright colours or numerous animations).

For the ASD module, white was used as a background colour for most of the buttons and three main options were differentiated through different colours: red for buttons whose actions were to exit or return to the previous page; yellow for buttons that featured instructions for different activities; and green for the help buttons. Other categorisations were not used because the project’s professionals reported that the same colour code was not always employed. Thus, the software allowed customisation of colours to suit the code used by each person.

### 3.2. Results from Observation

**Figure 4:**
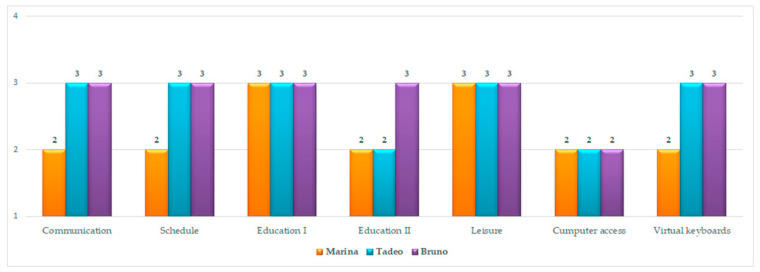
Scores obtained in the observation of the children’s interactions with the software.

Finally, Marina, Tadeo and Bruno obtained scores ranging from 2 (38%) to 3 (62%). Essentially, the children performed the activities requested in the testing session of the ASD Module with only verbal prompting or without assistance (Figure 4).

In addition, the observers qualitatively recorded aspects of the children’s interaction with the ASD Module:

- The children did not show behaviours of discomfort, rejection or opposition to the application or the tested interface.
- Marina, Tadeo and Bruno were involved in the task. In the exploration session, repetitive interactions with the software were observed, such as repetitive pressing of buttons or entering and exiting the different keyboards without apparent functionality.
- They showed technological competence to access and use the software and did not require physical support to operate the application.
- The need to personalise some images with photographs of the real environment of each child was detected (especially in the communication and schedule sections).
- The needs to reduce and customise the content options according to each child, their interests and needs, were detected.

## 4. Discussion

The purpose of this work was to identify and describe the recommended elements to support graphical user interface design for children with ASD. The thematic categories provided a guide with suggestions and some concerns for designing technology for children with autism from the design and development experience of the ASD Module.

The need to share the results of studies on the design and development of technology for people with autism through suggestions, guidelines or the development of a theoretical framework has been identified in previous studies [22,28,59]. Despite the efforts to investigate these guidelines, the results have been general or heterogeneous assumptions. No immovable guidelines were established in this study, but it involved suggestions based on the experience of this project (Table 2).

The ASD Module has been defined as a digital support that enables children with autism to engage and participate in the occupations they need, want, or are expected to perform. This study’s participants highlighted the areas of communication, leisure/play, time and activities management and education as those occupations in which children with ASD could benefit from the use of a digital support. These results are consistent with previous studies on the use, potential and desired outcomes of technology in the daily lives of people with autism [4,7,22,35,47,48,49,60]. Aspects such as creative or cognitive skills were not identified as priorities by stakeholders in this study [22].

One of the premises supported by almost all the literature is the need for customisation of technology [18,22,32,33,35,37,39,40,41,42,44,45,46,47,48,49]. In this study, the elements identified as customisable for each child were images, sound, reinforcement, text, colours, screen layout and presentation, and content.

The need to involve children with ASD, family members and professional interdisciplinary team members in the design of technology emerged from interviews and observations, as previously described in several publications [22,33,38,42,46,47,48,49]. Several authors have implemented studies to capture the perspectives on technology design of end-users and stakeholders in participatory design from a qualitative or mixed approach [22,41,42,44,45,46,49]. It is also necessary to properly select knowledge extraction techniques to help children, relatives and professionals capture their perspectives [38].

The results obtained based on the need for a simple and predictable structure of the application have been transferred to aspects, such as images, audio and virtual keyboards, allowing the avoidance of unnecessary information [32,33,40,42,48].

Visual supports were identified as indispensable elements of the ASD Module, as well as in other studies and best practice guides [1,19,34,39,40,41,42,45,47,48]. ARASAAC pictograms were the visual aids most used by all study participants due to their adaptation to the Spanish context and the perception of a greater iconicity of the symbols [54]. It is recognised, however, that the use of images must be adapted to each child’s level of visual cognition [61,62].

Other results on the interface design of the ASD Module were related to the following elements: synthesised voice, colour as a visual cue, or left-right orientation of information on the screen. Regarding the use of synthesised voice, the participants recommended its use [63], with the option of being customisable with natural voice, if necessary. The use of colour as a visual cue has been recognised as a facilitator for information processing [40,49,64]. The participants reported no total agreement on the use of specific colours for this purpose, although the grammatical classification that emerged with the PCS symbols predominated. They also indicated a predilection in the presentation of information on the screen following a left to right orientation, and specified that with some children, it would be important to allow a vertical arrangement [43,49].

The most complex design decision for participants was related to the involvement of atypical sensory patterns of children with autism. It has been explained that people with ASD process visual information more easily, although it is generally assumed that it is increasingly necessary to evaluate the sensory style of each person. Similarly, it is understood that the simultaneous display of auditory and visual stimuli assists in learning, but in some cases, people with ASD need to be provided with information through their preferred sensory channels. Current studies are consistent with the concerns of the participants in this study. On one hand, it is recommended to use a combination of text, images and sound [18,34,36,41]; however, on the other hand, it is recommended to pay attention to the presence of auditory and visual hyper-reactivity patterns or difficulties in interpreting stimuli as a whole unit of meaning in children with autism. In the second case, studies suggest designing technology that avoids the use of this combination of stimuli, allowing the reduction or elimination of unnecessary images, sounds, music or text [32,33,39,40,42,44,48].

This study had some important limitations. Its main limitation was the small number of participants in the group of children with autism and relatives. Difficulties in recruiting more participants in the children’s group were related to the difficulty in involving minors in research studies due to ethical considerations and the difficulties in conducting participatory studies in children with ASD with persistent communication difficulties. Concerning this, the techniques to facilitate the extraction of information from the participating children were also identified as a limitation, since other studies achieved the successful participation of children with these difficulties [47]. Also, the non-participation of adults with autism as a participants’ group is considered a limitation. This group could contribute with their perception of the use and preferences of technology as well.

Possible future lines of research should focus on the need for mixed method studies to gain in-depth understanding of the stakeholders’ perspectives on the technology design process and related experimental studies to confirm the results on interface design suggestions. Further discussion is needed regarding the aspect of generalising children’s learning using technology in their natural contexts in order to study the true impact on children’s daily lives.

## 5. Conclusions

The ASD Module is provided as an example available to children with autism, families and professionals so that, through the customisation of its necessary aspects, it could be turned into an assistive technology or digital support.

The results of this study reflect the meaning and perceptions of the features considered important in the user interface design for children with autism, from the perspective of the stakeholders.

The customisation of technology emerges throughout the participants’ speeches with strong intensity. The clarification of the principles and reasons for making decisions on the design of interface elements of a computer application resulted in interesting suggestions for practice and research in this area.

These suggested guiding principles for technology design provide useful information for researchers, developers, social and healthcare professionals and families, to offer alternatives for children with ASD and facilitate the understanding of daily life. Moreover, further research is necessary to develop participatory technology designs and validate these results.

## Figures and Tables

**Table 1:** Characteristics of participants.

Participant Demographics	Professionals: ASD	Professionals: Technology	Family Members	Children with ASD
Sample (*n* [%])	20 (51.3)	13 (33.3)	3 (7.7)	3 (7.7)
Age (years)				
Mean (SD)	41.6 (10.70)	34.2 (11.72)	57.0 (4.00)	12.0 (1.00)
Gender				
Female (*n* [%])	17 (43.6)	5 (12.8)	3 (7.7)	2 (5.1)
Male (*n* [%])	3 (7.7)	8 (20.5)	0	1 (2.6)
Expertise (years)				
Mean (SD)	16.0 (8.50)	4.9 (4.21)	NA	NA

Note: ASD = autism spectrum disorder; SD = standard deviation; NA = not applicable.

**Table 2:** Suggested recommendations to support interface design for children with autism.

1. The user should have the possibility to customise all relevant aspects
2. The software and its contents should be based on the person’s abilities, needs, and interests
3. The design of the interface and the information displayed should be simplified
4. Consider the use of images to display information
5. The images should convey the meaning of the real element
6. The images should allow personalisation according to users’ level of visual cognition.
7. The image should be accompanied by the written word
8. Speech synthesis is used to facilitate communication or as reinforcement to the command
9. The information can be displayed in a multimodal way (visual and auditory) and will be adapted according to the sensory style preferred by each child
10. The background colour should be used to facilitate the information processing

## Data Availability

The data presented in this study are available on request from the corresponding author (laura.nieto@udc.es). The data are not publicly available due to the need to assure participant confidentiality.

## Appendix A

### Appendix A1. Semi-Structured Interview Guide I

- Based on your personal and/or professional experience, what is your opinion about the use of technology in the daily life of people with ASD?
- What options would you like the application to include based on appropriate options to facilitate the daily life of a child with autism? Can you give specific examples of these options?
- What characteristics should the program have? Can you give specific examples of these features?

### Appendix A2. Semi-Structured Interview Guide II

- Demonstration and test of ASD Module
- What do you think about the contents of the ASD Module, in relation to the abilities and/or needs of children with ASD? Would you add or delete any of the contents?
- Which aspects or sections of the ASD Module would you highlight?
- How would you evaluate the ASD Module interface’s characteristics in terms of its usability for children with ASD (keyboard layout; button design and layout on the screen; use of colour in images, button background, and screen background; text: font type, size, location of the text concerning the image, among other characteristics; use and functionality of images; use and functionality of voice [synthesised or natural]; use, functionality, and modality of reinforcements)?
- What applicability do you think the ASD Module can have in the daily life of children with autism? Can you give specific examples?
- What contribution can the ASD Module make in comparison to the traditional methods used in the intervention with children with ASD?
- How would you define the ASD Module?
- What is your overall assessment of the ASD Module? What do you suggest as strengths and areas for improvement?

## Appendix B

**Table A1:** Summary of Themes, sub-themes, and software implications.

Themes	Subthemes	Implications for ASD Module
The user has the possibility to customise all relevant aspects	Adaptation, personalisation, and customisation is essential It ′depends″ on the child Diversity across the spectrum Each child is unique	ASD Module as an example Customisation of all elements
The software and its contents are based on the person’s abilities, needs, and interests	Consider individual abilities, needs, interests, and motivation Tehnology for daily life Communication, leisure/play, time and activities management, and education are relevant for children with autism Interest and motivation as a key point Predilection for technology	ASM Module as digital support for daily life ASD Module’s contents: schedule, education, leisure, communication, and computer access Customisation of contents
The design of the interface is simple, and the information displayed is simplified	Differences in information processing Simplifying information Little information Information in large size Consistent distribution Left-right organisation	ASD Module keyboards configuration is simple (whole display area, grid pattern, left-right axis). Customisation
Use of images to display information	Ability to process visual information Visual supports as a ley element	ASD Module was based on the use of image
Images convey the meaning of the real element	Iconicity of images Realistic and representative symbols ARASAAC pictograms The transition from PCS to ARASAAC pictograms	ASD Module includes an extensive collection of ARASAAC pictograms
The use of images allows users to adapt according to their level of visual cognition	Adaptation to the level of visual cognition Concern about the generalised use of pictograms Possible hierarchy Need for evaluation	ASD Module allows users to cus-tomise the program using other symbol sets
The image is accompanied by the written word	Combination of visual and written information Written word Bottom of image Capital letters and serif type style Lowercase and linked typeface Customisation	The primary source of information is the combination of image and writ-ten word at the bottom of the image
Speech synthesis is used to facilitate communication or as reinforcement to the command	Use of synthesised voice AAC system Auditory reinforcement of information Simple information Auditory hyper-reactivity Customisation: digitised voice	ASD Module buttons include a voice command using speech synthesis Messages are simple, and specific They could be replaced by the digitised voice
The information is displayed in a multimodal way (visual and auditory) and is adapted ac-cording to the sensory style preferred by each child	Facilitate learning Multimodal way: visual and auditory stimuli Sensory preferences Customisation	Multimodal presentation for buttons is used to provide the information through the image, written word, and speech synthesis Possibility to delete or add infor-mation (preferred sensory style)
The background colour is used to facilitate the information processing	Colour as a facilitator of learning Classifications of PCS symbols Adaptation to the needs Colour contrast Soft colour tones Consistency	ASD Module use white as back-ground and tree main options for some actions (red, yellow, and green) Possibility of customisation
